# 2,4,4-Tris(benzyl­sulfan­yl)-1,1-dichloro-3-nitro­buta-1,3-diene

**DOI:** 10.1107/S1600536808043419

**Published:** 2009-01-10

**Authors:** Goksin Aydinli, Cigdem Sayil, Cemil Ibis

**Affiliations:** a Istanbul University, Faculty of Engineering, Department of Chemistry, 34320 Avcilar-Istanbul, Turkey

## Abstract

In the title compound, C_25_H_21_Cl_2_NO_2_S_3_, the three phenyl rings are inclined to each other at dihedral angles of 68.4 (1), 79.5 (1) and 37.0 (1)°.

## Related literature

The C—C bond lengths of the butadiene chain agree well with corresponding distances in a similar compound (Surange *et al.*, 1997 ▶). For the biological activity of halogenobutadienes containing chlorine, see: Kalatskaya & Malama (1986 ▶). For the structures of nitro­butadienes, see: Ibis *et al.* (2006*a* ▶,*b* ▶). For the synthesis, see: Ibis & Aydinli (1999 ▶). For weighting schemes, see: Carruthers & Watkin (1979 ▶).
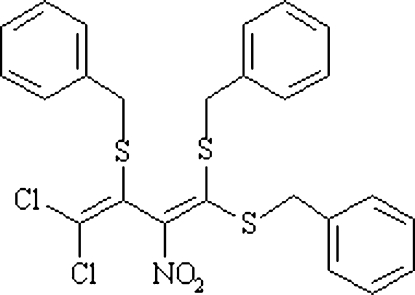


## Experimental

### 

#### Crystal data

C_25_H_21_Cl_2_NO_2_S_3_
*M* *_r_* = 534.53
Triclinic, 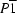

*a* = 10.1595 (10) Å
*b* = 11.5706 (10) Å
*c* = 12.5451 (2)
α = 74.887 (6)°
β = 69.259 (5)°
γ = 69.344 (5)°
*V* = 1274.83 (2) Å^3^
*Z* = 2
Mo *K*α radiation
μ = 0.52 mm^−1^
*T* = 293 K
0.60 × 0.60 × 0.10 mm

#### Data collection

Rigaku R-AXIS diffractometer
Absorption correction: multi-scan (*ABSCOR*; Higashi, 1995 ▶) *T*_min_ = 0.731, *T*_max_ = 0.949
101262 measured reflections
7512 independent reflections
7264 reflections with *F* ^2^ > 2.0σ(*F* ^2^)
*R*_int_ = 0.024

#### Refinement

*R*[*F* ^2^ > 2σ(*F* ^2^)] = 0.066
*wR*(*F* ^2^) = 0.045
*S* = 1.20
6957 reflections
319 parameters
H atoms treated by a mixture of independent and constrained refinment
Δρ_max_ = 0.31 e Å^−3^
Δρ_min_ = −0.34 e Å^−3^

### 

Data collection: *PROCESS* (Rigaku, 1996 ▶); cell refinement: *PROCESS*; data reduction: *CrystalStructure* (Rigaku/MSC, 2003 ▶); program(s) used to solve structure: *SIR92* (Altomare *et al.*, 1994 ▶); program(s) used to refine structure: *CRYSTALS* (Betteridge *et al.*, 2003 ▶); molecular graphics: *ORTEP-3* (Farrugia, 1997 ▶); software used to prepare material for publication: *CrystalStructure* (Rigaku/MSC, 2003 ▶).

## Supplementary Material

Crystal structure: contains datablocks global, I. DOI: 10.1107/S1600536808043419/xu2466sup1.cif

Structure factors: contains datablocks I. DOI: 10.1107/S1600536808043419/xu2466Isup2.hkl

Additional supplementary materials:  crystallographic information; 3D view; checkCIF report

## Figures and Tables

**Fig. 1.**
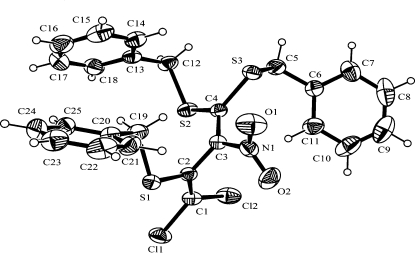
The molecular structure of the title compound. Displacement ellipsoids are drawn at the 30% probability level.

## supplementary crystallographic information

### Comment

There are numerous publications devoted to the synthesis of halogenobutadienes containing atom of chlorine. They possess a broad spectrum of useful properties: they are employed as monomers for the preparation of valuable polymers and copolymers resistant to heat, light, chemical corrosion and so on. And they show algicidal, bactericidal fungicidal activities (Kalatskaya & Malama, 1986). A number of halogenobutadienes manifest high antitumour activity. However, there are a few reports on the crystal structures of nitrobutadiene compounds (Ibis *et al.*, 2006a,b). It is the first publication about single-crystal structure of 1,1,3-tris(arylthio)nitrobutadiene derivative. The title compound was synthesized from 2-nitropentachlorobutadiene and benzyl mercaptan (Ibis & Aydinli, 1999). It is note that, our spectroscopic data are in accordance with reported by this article but apparently, title compound is not a 1,1,4-substituted, but a 1,1,3-substituted regioisomer instead. This indication was proven by X-ray analysis newly. Crystallographic analysis was carried out and the results are presented in this paper.

The molecular structure of the title compound is shown in Fig. 1. The three phenyl rings are inclined with the butadiene group at angles of 85.9 (1), 61.9 (1), 81.4 (1)°, respectively. The butadiene unit has assumed a configuration close to *cisoid*, but is not completely planar; torsion angle of C4—C3—C2—C1 is 75.3 (2)°.

### Experimental

2-Nitropentachlorobutadiene (2.0 g, 7.37 mmol) and benzyl mercaptan (2.74 g, 22.11 mmol) were mixed in ethanol(30 ml), 2 g of NaOH (in 10 ml of water) was added at room temperature. The mixture was stirred for 2–3 h. Chloroform was added to the reaction mixture. The organic layer was separated and washed with water (4 *x* 30 ml) and dried with Na_2_SO_4_. After the solvent had evaporated, the residue was purified by column chromatography on silica gel. Yellow crystals of (I) suitable for X-ray diffraction analysis were obtained by slow evaporation of an ethanol at room temperature (yield: 0.76 g, 20%; m.p. 357–358 K).

### Refinement

H atoms were treated as riding, with C—H = 0.95 (6)Å and *U*_iso_(H) = 1.2*U*_eq_(C). Refinement used 6597 reflections with F^2^ >3.0σ(F^2^).

### Crystal data

**Table:**

C_25_H_21_Cl_2_NO_2_S_3_	*Z* = 2
*M**_r_* = 534.53	*F*(000) = 552.00
Triclinic, *P*1	*D*_x_ = 1.392 Mg m^−^^3^
Hall symbol: -P 1	Mo *K*α radiation, λ = 0.7107 Å
*a* = 10.1595 (10) Å	Cell parameters from 10024 reflections
*b* = 11.5706 (10) Å	θ = 2.2–30.5°
*c* = 12.5451 Å	µ = 0.52 mm^−^^1^
α = 74.887 (6)°	*T* = 293 K
β = 69.259 (5)°	Prism, yellow
γ = 69.344 (5)°	0.60 × 0.60 × 0.10 mm
*V* = 1274.83 (2) Å^3^	

### Data collection

**Table:**

Rigaku R-AXIS diffractometer	7264 reflections with *F*^2^ > 2.0σ(*F*^2^)
Detector resolution: 10.00 pixels mm^-1^	*R*_int_ = 0.024
ω scans	θ_max_ = 30.3°
Absorption correction: multi-scan (*ABSCOR*; Higashi, 1995)	*h* = −14→14
*T*_min_ = 0.731, *T*_max_ = 0.949	*k* = −16→16
101262 measured reflections	*l* = −17→17
7512 independent reflections	

### Refinement

**Table:**

Refinement on *F*	All H-atom parameters refined
*R*[*F*^2^ > 2σ(*F*^2^)] = 0.066	Chebychev polynomial *w*ith 3 parameters (Carruthers & Watkin, 1979) 1.8080 -4.9818 -0.4430
*wR*(*F*^2^) = 0.045	(Δ/σ)_max_ < 0.001
*S* = 1.20	Δρ_max_ = 0.31 e Å^−^^3^
6957 reflections	Δρ_min_ = −0.34 e Å^−^^3^
319 parameters	

### Special details

**Table:**

Geometry. ENTER SPECIAL DETAILS OF THE MOLECULAR GEOMETRY
Refinement. Refinement using all reflections. The weighted R-factor(wR), goodness of fit (S) and R-factor (gt) are based on F, with F set to zero for negative F. The threshold expression of F^2^ > 2.0 sigma(F^2^) is used only for calculating R-factor (gt).

### Fractional atomic coordinates and isotropic or equivalent isotropic displacement parameters (Å^2^)

**Table:**

	*x*	*y*	*z*	*U*_iso_*/*U*_eq_	
S(1)	0.26233 (3)	−0.27301 (2)	0.14860 (2)	0.06102 (8)	
S(2)	0.51264 (3)	−0.16364 (2)	−0.00818 (2)	0.05869 (7)	
S(3)	0.32274 (3)	−0.18195 (2)	0.43976 (2)	0.05472 (7)	
Cl(2)	0.46317 (4)	−0.52367 (3)	0.29977 (3)	0.08279 (10)	
Cl(1)	0.29918 (3)	−0.45732 (3)	0.52456 (2)	0.07867 (9)	
O(2)	0.69150 (9)	−0.31748 (10)	0.25152 (9)	0.1049 (3)	
C(3)	0.46483 (10)	−0.24924 (8)	0.22255 (7)	0.0452 (2)	
N(1)	0.60515 (10)	−0.23115 (9)	0.21164 (7)	0.0599 (3)	
C(6)	0.06852 (11)	−0.03451 (9)	0.12175 (8)	0.0554 (3)	
C(2)	0.38853 (10)	−0.29043 (8)	0.34376 (8)	0.0472 (2)	
C(12)	0.53745 (12)	−0.27616 (9)	−0.09776 (8)	0.0656 (3)	
C(4)	0.41789 (10)	−0.22722 (8)	0.12919 (8)	0.0467 (2)	
O(1)	0.62431 (12)	−0.12993 (9)	0.16884 (9)	0.1054 (4)	
C(5)	0.15905 (13)	−0.14498 (10)	0.06169 (9)	0.0679 (3)	
C(19)	0.2622 (1)	−0.03352 (10)	0.35006 (10)	0.0723 (3)	
C(25)	0.09262 (11)	0.13681 (10)	0.46338 (9)	0.0611 (3)	
C(1)	0.38339 (11)	−0.40762 (9)	0.38298 (8)	0.0545 (3)	
C(24)	0.0636 (1)	0.22953 (11)	0.52495 (11)	0.0740 (4)	
C(7)	0.11962 (13)	0.06683 (10)	0.10021 (10)	0.0685 (4)	
C(13)	0.68084 (11)	−0.37659 (8)	−0.10637 (8)	0.0571 (3)	
C(11)	−0.0680 (1)	−0.03113 (11)	0.19867 (10)	0.0769 (4)	
C(22)	0.3176 (2)	0.18959 (12)	0.49366 (13)	0.0873 (5)	
C(20)	0.23412 (11)	0.06770 (9)	0.41576 (8)	0.0562 (3)	
C(21)	0.34768 (12)	0.09577 (10)	0.43161 (11)	0.0728 (4)	
C(23)	0.1763 (2)	0.25476 (11)	0.54108 (12)	0.0851 (5)	
C(18)	0.7057 (2)	−0.45940 (10)	−0.00968 (10)	0.0802 (4)	
C(8)	0.0382 (2)	0.16857 (11)	0.1559 (1)	0.0899 (5)	
C(14)	0.7909 (2)	−0.38460 (12)	−0.21024 (13)	0.0853 (4)	
C(10)	−0.1501 (2)	0.0705 (2)	0.25525 (12)	0.1016 (5)	
C(9)	−0.0961 (2)	0.1699 (1)	0.23337 (13)	0.1019 (6)	
C(15)	0.9252 (2)	−0.4770 (2)	−0.2127 (2)	0.1179 (7)	
C(16)	0.9462 (2)	−0.5565 (2)	−0.1155 (2)	0.1120 (7)	
C(17)	0.8397 (2)	−0.54888 (13)	−0.0147 (2)	0.1095 (6)	
H(1)	0.22499 (13)	−0.12013 (10)	−0.00917 (9)	0.0843 (11)*	
H(2)	0.09310 (13)	−0.17564 (10)	0.04664 (9)	0.0847 (10)*	
H(3)	0.21293 (13)	0.06651 (10)	0.04609 (10)	0.0850 (11)*	
H(4)	0.0744 (2)	0.23844 (11)	0.1398 (1)	0.1130 (11)*	
H(5)	−0.1524 (2)	0.2386 (1)	0.27395 (13)	0.1181 (11)*	
H(6)	−0.2439 (2)	0.0719 (2)	0.30899 (12)	0.1091 (11)*	
H(7)	−0.1066 (1)	−0.09929 (11)	0.21298 (10)	0.0912 (10)*	
H(8)	0.46109 (12)	−0.31533 (9)	−0.06329 (8)	0.0781 (10)*	
H(9)	0.53273 (12)	−0.23380 (9)	−0.17275 (8)	0.0787 (10)*	
H(10)	0.7753 (2)	−0.32952 (12)	−0.27839 (13)	0.0981 (11)*	
H(11)	1.0029 (2)	−0.4859 (2)	−0.2826 (2)	0.1339 (12)*	
H(12)	1.0384 (2)	−0.6175 (2)	−0.1177 (2)	0.1381 (13)*	
H(13)	0.8561 (2)	−0.60471 (13)	0.0529 (2)	0.1335 (12)*	
H(14)	0.6289 (2)	−0.45517 (10)	0.06095 (10)	0.0920 (11)*	
H(15)	0.3368 (1)	−0.02515 (10)	0.27928 (10)	0.0850 (11)*	
H(16)	0.1750 (1)	−0.02808 (10)	0.33470 (10)	0.0850 (10)*	
H(17)	0.44699 (12)	0.05170 (10)	0.39823 (11)	0.0860 (10)*	
H(18)	0.3949 (2)	0.20947 (12)	0.50400 (13)	0.1159 (11)*	
H(19)	0.1562 (2)	0.31794 (11)	0.58487 (12)	0.1074 (11)*	
H(20)	−0.0344 (1)	0.27810 (11)	0.55578 (11)	0.0867 (10)*	
H(21)	0.01245 (11)	0.12000 (10)	0.45437 (9)	0.0742 (10)*	

### Atomic displacement parameters (Å^2^)

**Table:**

	*U*^11^	*U*^22^	*U*^33^	*U*^12^	*U*^13^	*U*^23^
S(1)	0.0677 (2)	0.0591 (1)	0.0658 (2)	−0.02160 (13)	−0.03755 (13)	0.00494 (12)
S(2)	0.0776 (2)	0.04780 (13)	0.04488 (13)	−0.01718 (12)	−0.01546 (12)	−0.00220 (10)
S(3)	0.0585 (2)	0.0586 (1)	0.04394 (12)	−0.01226 (12)	−0.01779 (11)	−0.00514 (10)
Cl(2)	0.1318 (3)	0.0530 (2)	0.0759 (2)	−0.0324 (2)	−0.0456 (2)	−0.00051 (13)
Cl(1)	0.0866 (2)	0.0846 (2)	0.0624 (2)	−0.0428 (2)	−0.0246 (2)	0.0219 (1)
O(2)	0.0538 (5)	0.1180 (7)	0.1299 (8)	−0.0190 (5)	−0.0382 (5)	0.0103 (6)
C(3)	0.0499 (5)	0.0443 (5)	0.0433 (5)	−0.0151 (4)	−0.0167 (4)	−0.0032 (4)
N(1)	0.0611 (6)	0.0762 (6)	0.0486 (5)	−0.0301 (5)	−0.0156 (4)	−0.0060 (4)
C(6)	0.0617 (6)	0.0606 (6)	0.0490 (5)	−0.0159 (5)	−0.0324 (5)	0.0034 (4)
C(2)	0.0495 (5)	0.0522 (5)	0.0437 (5)	−0.0175 (4)	−0.0209 (4)	0.0006 (4)
C(12)	0.0761 (7)	0.0714 (7)	0.0485 (5)	−0.0072 (6)	−0.0254 (5)	−0.0173 (5)
C(4)	0.0551 (5)	0.0391 (4)	0.0458 (5)	−0.0092 (4)	−0.0202 (4)	−0.0046 (4)
O(1)	0.1254 (8)	0.1100 (7)	0.1164 (8)	−0.0819 (7)	−0.0554 (6)	0.0196 (6)
C(5)	0.0762 (7)	0.0702 (7)	0.0646 (6)	−0.0093 (6)	−0.0437 (6)	−0.0063 (5)
C(19)	0.0924 (8)	0.0576 (6)	0.0612 (6)	−0.0034 (6)	−0.0362 (6)	−0.0061 (5)
C(25)	0.0547 (6)	0.0614 (6)	0.0680 (7)	−0.0163 (5)	−0.0222 (5)	−0.0060 (5)
C(1)	0.0628 (6)	0.0574 (5)	0.0510 (5)	−0.0262 (5)	−0.0259 (5)	0.0047 (4)
C(24)	0.0758 (8)	0.0658 (7)	0.0726 (7)	−0.0105 (6)	−0.0203 (6)	−0.0137 (6)
C(7)	0.0747 (7)	0.0637 (7)	0.0750 (7)	−0.0182 (6)	−0.0414 (6)	0.0018 (6)
C(13)	0.0680 (6)	0.0514 (5)	0.0573 (6)	−0.0148 (5)	−0.0236 (5)	−0.0131 (5)
C(11)	0.0743 (8)	0.0828 (9)	0.0686 (7)	−0.0280 (7)	−0.0222 (7)	0.0061 (7)
C(22)	0.0925 (10)	0.0800 (8)	0.1155 (11)	−0.0402 (8)	−0.0592 (9)	0.0043 (8)
C(20)	0.0606 (6)	0.0549 (5)	0.0545 (6)	−0.0151 (5)	−0.0243 (5)	−0.0022 (5)
C(21)	0.0542 (7)	0.0724 (7)	0.0888 (8)	−0.0151 (5)	−0.0306 (6)	0.0018 (6)
C(23)	0.1182 (12)	0.0600 (7)	0.0887 (9)	−0.0220 (8)	−0.0474 (9)	−0.0114 (6)
C(18)	0.1006 (10)	0.0645 (7)	0.0645 (7)	−0.0003 (7)	−0.0297 (7)	−0.0169 (6)
C(8)	0.1181 (12)	0.0659 (8)	0.0985 (10)	−0.0148 (8)	−0.0651 (10)	−0.0030 (7)
C(14)	0.0885 (9)	0.0708 (8)	0.0861 (9)	−0.0348 (7)	0.0043 (7)	−0.0205 (7)
C(10)	0.0850 (10)	0.1219 (13)	0.0632 (8)	−0.0115 (9)	−0.0092 (7)	−0.0005 (9)
C(9)	0.137 (2)	0.0843 (10)	0.0741 (9)	0.0060 (10)	−0.0504 (10)	−0.0200 (8)
C(15)	0.0842 (11)	0.0939 (11)	0.155 (2)	−0.0424 (10)	0.0290 (11)	−0.0525 (12)
C(16)	0.0712 (10)	0.0860 (11)	0.189 (2)	−0.0078 (8)	−0.0403 (12)	−0.0548 (13)
C(17)	0.1273 (13)	0.0714 (9)	0.135 (2)	0.0171 (9)	−0.0780 (13)	−0.0317 (9)

### Geometric parameters (Å, °)

**Table:**

S(1)—C(4)	1.757 (1)	C(24)—C(23)	1.367 (3)
S(1)—C(5)	1.832 (1)	C(24)—H(20)	0.950 (2)
S(2)—C(12)	1.831 (1)	C(7)—C(8)	1.375 (2)
S(2)—C(4)	1.7610 (9)	C(7)—H(3)	0.950 (2)
S(3)—C(2)	1.760 (1)	C(13)—C(18)	1.376 (1)
S(3)—C(19)	1.825 (1)	C(13)—C(14)	1.384 (2)
Cl(2)—C(1)	1.707 (1)	C(11)—C(10)	1.381 (2)
Cl(1)—C(1)	1.7192 (9)	C(11)—H(7)	0.950 (2)
O(2)—N(1)	1.204 (1)	C(22)—C(21)	1.384 (2)
C(3)—N(1)	1.467 (2)	C(22)—C(23)	1.359 (2)
C(3)—C(2)	1.479 (1)	C(22)—H(18)	0.950 (3)
C(3)—C(4)	1.348 (2)	C(20)—C(21)	1.392 (2)
N(1)—O(1)	1.207 (1)	C(21)—H(17)	0.950 (1)
C(6)—C(5)	1.493 (1)	C(23)—H(19)	0.950 (2)
C(6)—C(7)	1.372 (2)	C(18)—C(17)	1.381 (2)
C(6)—C(11)	1.375 (1)	C(18)—H(14)	0.950 (2)
C(2)—C(1)	1.329 (1)	C(8)—C(9)	1.364 (2)
C(12)—C(13)	1.498 (1)	C(8)—H(4)	0.950 (2)
C(12)—H(8)	0.950 (2)	C(14)—C(15)	1.401 (2)
C(12)—H(9)	0.950 (1)	C(14)—H(10)	0.950 (2)
C(5)—H(1)	0.950 (1)	C(10)—C(9)	1.368 (3)
C(5)—H(2)	0.950 (2)	C(10)—H(6)	0.950 (2)
C(19)—C(20)	1.494 (2)	C(9)—H(5)	0.950 (2)
C(19)—H(15)	0.950 (2)	C(15)—C(16)	1.355 (3)
C(19)—H(16)	0.950 (2)	C(15)—H(11)	0.950 (3)
C(25)—C(24)	1.372 (2)	C(16)—C(17)	1.341 (3)
C(25)—C(20)	1.376 (1)	C(16)—H(12)	0.950 (2)
C(25)—H(21)	0.950 (2)	C(17)—H(13)	0.950 (3)
			
S(1)···S(2)	3.0605 (4)	C(21)···H(10)^ii^	3.029 (2)
S(1)···Cl(2)	3.4452 (4)	C(21)···H(15)	2.697 (2)
S(1)···C(3)	2.656 (1)	C(21)···H(16)	3.287 (2)
S(1)···C(6)	2.7852 (9)	C(21)···H(18)	2.040 (2)
S(1)···C(2)	3.076 (1)	C(21)···H(19)	3.239 (2)
S(1)···C(12)	3.3517 (9)	C(21)···H(21)	3.231 (2)
S(1)···C(5)	1.832 (1)	C(23)···C(25)	2.374 (2)
S(1)···C(1)	3.377 (1)	C(23)···C(20)	2.779 (2)
S(1)···C(11)	3.505 (1)	C(23)···C(21)	2.380 (2)
S(1)···C(16)^i^	3.542 (2)	C(23)···H(10)^ii^	3.085 (2)
S(1)···C(17)^i^	3.562 (3)	C(23)···H(17)	3.231 (2)
S(1)···H(1)	2.329 (1)	C(23)···H(18)	1.999 (2)
S(1)···H(2)	2.302 (1)	C(23)···H(20)	2.007 (2)
S(1)···H(7)	3.483 (1)	C(23)···H(21)	3.223 (2)
S(1)···H(8)	2.7500 (9)	C(18)···S(2)	3.291 (1)
S(1)···H(14)	3.506 (1)	C(18)···C(12)	2.490 (2)
S(2)···S(1)	3.0605 (4)	C(18)···C(4)	3.477 (1)
S(2)···C(3)	2.7284 (8)	C(18)···C(14)	2.383 (2)
S(2)···N(1)	3.048 (1)	C(18)···C(15)	2.725 (2)
S(2)···C(12)	1.831 (1)	C(18)···C(16)	2.353 (2)
S(2)···O(1)	2.987 (1)	C(18)···H(8)	2.662 (2)
S(2)···O(1)^ii^	3.5522 (9)	C(18)···H(8)^i^	3.358 (2)
S(2)···C(5)	3.323 (1)	C(18)···H(9)	3.258 (2)
S(2)···C(13)	2.7622 (9)	C(18)···H(10)	3.247 (2)
S(2)···C(18)	3.291 (1)	C(18)···H(12)	3.208 (2)
S(2)···H(1)	2.789 (1)	C(18)···H(13)	2.031 (2)
S(2)···H(3)	3.253 (1)	C(8)···C(6)	2.392 (2)
S(2)···H(3)^ii^	3.202 (2)	C(8)···C(11)	2.737 (2)
S(2)···H(8)	2.317 (1)	C(8)···C(10)	2.368 (2)
S(2)···H(9)	2.328 (1)	C(8)···H(2)^iv^	3.233 (3)
S(2)···H(14)	3.149 (1)	C(8)···H(3)	2.020 (2)
S(3)···Cl(1)	3.1514 (4)	C(8)···H(5)	2.021 (2)
S(3)···C(3)	2.7645 (9)	C(8)···H(6)	3.224 (2)
S(3)···N(1)	3.2600 (8)	C(8)···H(16)	3.075 (2)
S(3)···C(19)	1.825 (1)	C(8)···H(21)	3.566 (2)
S(3)···C(1)	2.689 (1)	C(14)···C(12)	2.503 (2)
S(3)···C(20)	2.674 (1)	C(14)···C(18)	2.383 (2)
S(3)···C(21)	3.284 (1)	C(14)···C(16)	2.395 (2)
S(3)···H(15)	2.332 (1)	C(14)···C(17)	2.764 (2)
S(3)···H(16)	2.335 (1)	C(14)···H(4)^ii^	2.963 (3)
S(3)···H(17)	3.222 (1)	C(14)···H(8)	3.125 (2)
Cl(2)···S(1)	3.4452 (4)	C(14)···H(9)	2.544 (2)
Cl(2)···Cl(1)	2.8664 (4)	C(14)···H(11)	2.056 (2)
Cl(2)···C(3)	3.073 (1)	C(14)···H(12)	3.254 (2)
Cl(2)···C(2)	2.683 (1)	C(14)···H(14)	3.238 (2)
Cl(2)···C(4)	3.5090 (9)	C(10)···C(6)	2.395 (2)
Cl(2)···H(9)^i^	3.552 (1)	C(10)···C(7)	2.735 (2)
Cl(2)···H(10)^i^	3.526 (2)	C(10)···C(8)	2.368 (2)
Cl(2)···H(14)	2.927 (1)	C(10)···H(1)^iv^	3.297 (2)
Cl(2)···H(18)^iii^	3.565 (1)	C(10)···H(2)^iv^	3.564 (2)
Cl(1)···S(3)	3.1514 (4)	C(10)···H(4)	3.225 (3)
Cl(1)···Cl(2)	2.8664 (4)	C(10)···H(5)	2.010 (3)
Cl(1)···C(2)	2.6818 (9)	C(10)···H(7)	2.025 (2)
Cl(1)···H(19)^iii^	3.210 (2)	C(10)···H(16)	3.505 (2)
O(2)···C(3)	2.285 (1)	C(9)···C(6)	2.762 (2)
O(2)···C(2)	2.810 (1)	C(9)···C(7)	2.370 (2)
O(2)···C(4)	3.363 (2)	C(9)···C(11)	2.377 (2)
O(2)···O(1)	2.129 (1)	C(9)···H(2)^iv^	3.485 (2)
O(2)···C(1)	3.370 (1)	C(9)···H(3)	3.225 (2)
O(2)···H(14)	3.527 (2)	C(9)···H(4)	2.013 (3)
C(3)···S(1)	2.656 (1)	C(9)···H(6)	2.018 (3)
C(3)···S(2)	2.7284 (8)	C(9)···H(7)	3.232 (2)
C(3)···S(3)	2.7645 (9)	C(9)···H(16)	3.310 (2)
C(3)···Cl(2)	3.073 (1)	C(9)···H(21)	3.181 (2)
C(3)···O(2)	2.285 (1)	C(15)···C(13)	2.391 (2)
C(3)···O(1)	2.303 (2)	C(15)···C(18)	2.725 (2)
C(3)···C(19)	3.025 (1)	C(15)···C(17)	2.349 (3)
C(3)···C(1)	2.462 (1)	C(15)···H(4)^ii^	3.128 (3)
C(3)···H(14)	3.145 (1)	C(15)···H(10)	2.057 (2)
C(3)···H(15)	2.617 (1)	C(15)···H(12)	2.007 (3)
C(3)···H(16)	3.290 (1)	C(15)···H(13)	3.206 (3)
N(1)···S(2)	3.048 (1)	C(16)···S(1)^i^	3.542 (2)
N(1)···S(3)	3.2600 (8)	C(16)···C(13)	2.749 (2)
N(1)···C(2)	2.439 (1)	C(16)···C(18)	2.353 (2)
N(1)···C(4)	2.449 (2)	C(16)···C(14)	2.395 (2)
N(1)···C(19)	3.542 (1)	C(16)···H(2)^i^	3.121 (2)
N(1)···C(1)	3.375 (1)	C(16)···H(4)^ii^	3.549 (2)
N(1)···H(3)^ii^	3.541 (1)	C(16)···H(10)	3.253 (2)
N(1)···H(14)	3.484 (2)	C(16)···H(11)	1.993 (3)
N(1)···H(15)	2.943 (1)	C(16)···H(13)	1.997 (3)
C(6)···S(1)	2.7852 (9)	C(16)···H(14)	3.209 (2)
C(6)···C(6)^iv^	3.587 (2)	C(17)···S(1)^i^	3.562 (3)
C(6)···C(4)	3.482 (1)	C(17)···C(13)	2.398 (2)
C(6)···C(5)^iv^	3.584 (2)	C(17)···C(14)	2.764 (2)
C(6)···C(8)	2.392 (2)	C(17)···C(15)	2.349 (3)
C(6)···C(10)	2.395 (2)	C(17)···H(2)^i^	3.118 (2)
C(6)···C(9)	2.762 (2)	C(17)···H(4)^vi^	3.485 (3)
C(6)···H(1)	2.023 (1)	C(17)···H(11)	3.194 (3)
C(6)···H(1)^iv^	3.463 (2)	C(17)···H(12)	1.983 (2)
C(6)···H(2)	1.991 (2)	C(17)···H(14)	2.030 (2)
C(6)···H(2)^iv^	3.167 (1)	H(1)···S(1)	2.329 (1)
C(6)···H(3)	2.016 (2)	H(1)···S(2)	2.789 (1)
C(6)···H(4)	3.247 (2)	H(1)···C(6)	2.023 (1)
C(6)···H(6)	3.252 (2)	H(1)···C(6)^iv^	3.463 (2)
C(6)···H(7)	2.022 (2)	H(1)···C(12)	3.013 (1)
C(6)···H(16)	3.243 (2)	H(1)···C(4)	2.822 (2)
C(2)···S(1)	3.076 (1)	H(1)···O(1)^ii^	3.558 (2)
C(2)···Cl(2)	2.683 (1)	H(1)···C(7)	2.566 (2)
C(2)···Cl(1)	2.6818 (9)	H(1)···C(11)	3.234 (1)
C(2)···O(2)	2.810 (1)	H(1)···C(11)^iv^	3.131 (2)
C(2)···N(1)	2.439 (1)	H(1)···C(10)^iv^	3.297 (2)
C(2)···C(4)	2.535 (1)	H(2)···S(1)	2.302 (1)
C(2)···O(1)	3.360 (1)	H(2)···C(6)	1.991 (2)
C(2)···C(19)	2.802 (1)	H(2)···C(6)^iv^	3.167 (1)
C(2)···H(15)	2.867 (1)	H(2)···C(7)	3.169 (2)
C(2)···H(16)	3.047 (1)	H(2)···C(7)^iv^	3.056 (2)
C(12)···S(1)	3.3517 (9)	H(2)···C(11)	2.575 (2)
C(12)···S(2)	1.831 (1)	H(2)···C(11)^iv^	3.398 (2)
C(12)···C(4)	2.794 (1)	H(2)···C(8)^iv^	3.233 (3)
C(12)···C(18)	2.490 (2)	H(2)···C(10)^iv^	3.564 (2)
C(12)···C(14)	2.503 (2)	H(2)···C(9)^iv^	3.485 (2)
C(12)···H(1)	3.013 (1)	H(2)···C(16)^i^	3.121 (2)
C(12)···H(10)	2.675 (2)	H(2)···C(17)^i^	3.118 (2)
C(12)···H(14)	2.630 (1)	H(3)···S(2)	3.253 (1)
C(4)···Cl(2)	3.5090 (9)	H(3)···S(2)^ii^	3.202 (2)
C(4)···O(2)	3.363 (2)	H(3)···N(1)^ii^	3.541 (1)
C(4)···N(1)	2.449 (2)	H(3)···C(6)	2.016 (2)
C(4)···C(6)	3.482 (1)	H(3)···C(4)	3.416 (1)
C(4)···C(2)	2.535 (1)	H(3)···O(1)^ii^	2.685 (1)
C(4)···C(12)	2.794 (1)	H(3)···C(5)	2.635 (2)
C(4)···O(1)	2.934 (2)	H(3)···C(11)	3.219 (2)
C(4)···C(5)	2.816 (2)	H(3)···C(8)	2.020 (2)
C(4)···C(19)	3.583 (2)	H(3)···C(9)	3.225 (2)
C(4)···C(1)	3.298 (1)	H(4)···C(6)	3.247 (2)
C(4)···C(13)	3.560 (1)	H(4)···C(7)	2.027 (2)
C(4)···C(18)	3.477 (1)	H(4)···C(13)^ii^	3.260 (2)
C(4)···H(1)	2.822 (2)	H(4)···C(14)^ii^	2.963 (3)
C(4)···H(3)	3.416 (1)	H(4)···C(10)	3.225 (3)
C(4)···H(8)	2.698 (2)	H(4)···C(9)	2.013 (3)
C(4)···H(9)	3.560 (1)	H(4)···C(15)^ii^	3.128 (3)
C(4)···H(14)	2.859 (1)	H(4)···C(16)^ii^	3.549 (2)
C(4)···H(15)	3.087 (2)	H(5)···C(7)	3.233 (2)
C(4)···H(16)	3.553 (1)	H(5)···C(11)	3.232 (2)
O(1)···S(2)	2.987 (1)	H(5)···C(8)	2.021 (2)
O(1)···S(2)^ii^	3.5522 (9)	H(5)···C(10)	2.010 (3)
O(1)···O(2)	2.129 (1)	H(6)···C(6)	3.252 (2)
O(1)···C(3)	2.303 (2)	H(6)···C(11)	2.033 (2)
O(1)···C(2)	3.360 (1)	H(6)···C(8)	3.224 (2)
O(1)···C(4)	2.934 (2)	H(6)···C(9)	2.018 (3)
O(1)···C(19)	3.528 (1)	H(7)···S(1)	3.483 (1)
O(1)···C(7)^ii^	3.540 (1)	H(7)···C(6)	2.022 (2)
O(1)···H(1)^ii^	3.558 (2)	H(7)···C(5)	2.656 (2)
O(1)···H(3)^ii^	2.685 (1)	H(7)···C(7)	3.221 (2)
O(1)···H(7)^v^	3.125 (2)	H(7)···C(10)	2.025 (2)
O(1)···H(15)	2.726 (1)	H(7)···C(9)	3.232 (2)
O(1)···H(17)	3.571 (2)	H(8)···S(1)	2.7500 (9)
C(5)···S(1)	1.832 (1)	H(8)···S(2)	2.317 (1)
C(5)···S(2)	3.323 (1)	H(8)···C(4)	2.698 (2)
C(5)···C(6)^iv^	3.584 (2)	H(8)···C(5)	3.114 (1)
C(5)···C(4)	2.816 (2)	H(8)···C(13)	2.002 (1)
C(5)···C(7)	2.488 (2)	H(8)···C(18)	2.662 (2)
C(5)···C(11)	2.498 (1)	H(8)···C(18)^i^	3.358 (2)
C(5)···H(3)	2.635 (2)	H(8)···C(14)	3.125 (2)
C(5)···H(7)	2.656 (2)	H(9)···S(2)	2.328 (1)
C(5)···H(8)	3.114 (1)	H(9)···Cl(2)^i^	3.552 (1)
C(19)···S(3)	1.825 (1)	H(9)···C(4)	3.560 (1)
C(19)···C(3)	3.025 (1)	H(9)···C(13)	2.026 (1)
C(19)···N(1)	3.542 (1)	H(9)···C(21)^ii^	3.232 (1)
C(19)···C(2)	2.802 (1)	H(9)···C(18)	3.258 (2)
C(19)···C(4)	3.583 (2)	H(9)···C(14)	2.544 (2)
C(19)···O(1)	3.528 (1)	H(10)···Cl(2)^i^	3.526 (2)
C(19)···C(25)	2.491 (1)	H(10)···C(12)	2.675 (2)
C(19)···C(21)	2.526 (2)	H(10)···C(25)^ii^	3.060 (2)
C(19)···H(17)	2.698 (2)	H(10)···C(24)^ii^	3.077 (2)
C(19)···H(21)	2.643 (1)	H(10)···C(13)	2.041 (2)
C(25)···C(19)	2.491 (1)	H(10)···C(22)^ii^	3.042 (2)
C(25)···C(22)	2.731 (2)	H(10)···C(20)^ii^	3.057 (2)
C(25)···C(21)	2.366 (2)	H(10)···C(21)^ii^	3.029 (2)
C(25)···C(23)	2.374 (2)	H(10)···C(23)^ii^	3.085 (2)
C(25)···H(10)^ii^	3.060 (2)	H(10)···C(18)	3.247 (2)
C(25)···H(15)	3.150 (1)	H(10)···C(15)	2.057 (2)
C(25)···H(16)	2.543 (2)	H(10)···C(16)	3.253 (2)
C(25)···H(17)	3.230 (2)	H(11)···C(13)	3.257 (2)
C(25)···H(19)	3.233 (2)	H(11)···C(14)	2.056 (2)
C(25)···H(20)	2.030 (2)	H(11)···C(16)	1.993 (3)
C(1)···S(1)	3.377 (1)	H(11)···C(17)	3.194 (3)
C(1)···S(3)	2.689 (1)	H(12)···C(18)	3.208 (2)
C(1)···O(2)	3.370 (1)	H(12)···C(14)	3.254 (2)
C(1)···C(3)	2.462 (1)	H(12)···C(15)	2.007 (3)
C(1)···N(1)	3.375 (1)	H(12)···C(17)	1.983 (2)
C(1)···C(4)	3.298 (1)	H(13)···C(13)	3.253 (2)
C(24)···C(22)	2.358 (2)	H(13)···C(18)	2.031 (2)
C(24)···C(20)	2.398 (1)	H(13)···C(8)^vi^	2.911 (2)
C(24)···C(21)	2.738 (1)	H(13)···C(9)^vi^	2.995 (2)
C(24)···H(10)^ii^	3.077 (2)	H(13)···C(15)	3.206 (3)
C(24)···H(18)	3.210 (2)	H(13)···C(16)	1.997 (3)
C(24)···H(19)	2.020 (3)	H(14)···S(1)	3.506 (1)
C(24)···H(21)	2.009 (2)	H(14)···S(2)	3.149 (1)
C(7)···O(1)^ii^	3.540 (1)	H(14)···Cl(2)	2.927 (1)
C(7)···C(5)	2.488 (2)	H(14)···O(2)	3.527 (2)
C(7)···C(11)	2.360 (2)	H(14)···C(3)	3.145 (1)
C(7)···C(10)	2.735 (2)	H(14)···N(1)	3.484 (2)
C(7)···C(9)	2.370 (2)	H(14)···C(12)	2.630 (1)
C(7)···H(1)	2.566 (2)	H(14)···C(4)	2.859 (1)
C(7)···H(2)	3.169 (2)	H(14)···C(13)	2.017 (1)
C(7)···H(2)^iv^	3.056 (2)	H(14)···C(14)	3.238 (2)
C(7)···H(4)	2.027 (2)	H(14)···C(16)	3.209 (2)
C(7)···H(5)	3.233 (2)	H(14)···C(17)	2.030 (2)
C(7)···H(7)	3.221 (2)	H(15)···S(3)	2.332 (1)
C(7)···H(15)	3.414 (2)	H(15)···C(3)	2.617 (1)
C(7)···H(16)	3.038 (2)	H(15)···N(1)	2.943 (1)
C(13)···S(2)	2.7622 (9)	H(15)···C(2)	2.867 (1)
C(13)···C(4)	3.560 (1)	H(15)···C(4)	3.087 (2)
C(13)···C(15)	2.391 (2)	H(15)···O(1)	2.726 (1)
C(13)···C(16)	2.749 (2)	H(15)···C(25)	3.150 (1)
C(13)···C(17)	2.398 (2)	H(15)···C(7)	3.414 (2)
C(13)···H(4)^ii^	3.260 (2)	H(15)···C(20)	2.019 (2)
C(13)···H(8)	2.002 (1)	H(15)···C(21)	2.697 (2)
C(13)···H(9)	2.026 (1)	H(16)···S(3)	2.335 (1)
C(13)···H(10)	2.041 (2)	H(16)···C(3)	3.290 (1)
C(13)···H(11)	3.257 (2)	H(16)···C(6)	3.243 (2)
C(13)···H(13)	3.253 (2)	H(16)···C(2)	3.047 (1)
C(13)···H(14)	2.017 (1)	H(16)···C(4)	3.553 (1)
C(11)···S(1)	3.505 (1)	H(16)···C(25)	2.543 (2)
C(11)···C(5)	2.498 (1)	H(16)···C(7)	3.038 (2)
C(11)···C(7)	2.360 (2)	H(16)···C(11)	3.476 (2)
C(11)···C(8)	2.737 (2)	H(16)···C(20)	2.028 (2)
C(11)···C(9)	2.377 (2)	H(16)···C(21)	3.287 (2)
C(11)···H(1)	3.234 (1)	H(16)···C(8)	3.075 (2)
C(11)···H(1)^iv^	3.131 (2)	H(16)···C(10)	3.505 (2)
C(11)···H(2)	2.575 (2)	H(16)···C(9)	3.310 (2)
C(11)···H(2)^iv^	3.398 (2)	H(17)···S(3)	3.222 (1)
C(11)···H(3)	3.219 (2)	H(17)···O(1)	3.571 (2)
C(11)···H(5)	3.232 (2)	H(17)···C(19)	2.698 (2)
C(11)···H(6)	2.033 (2)	H(17)···C(25)	3.230 (2)
C(11)···H(16)	3.476 (2)	H(17)···C(22)	2.027 (2)
C(22)···C(25)	2.731 (2)	H(17)···C(20)	2.040 (2)
C(22)···C(24)	2.358 (2)	H(17)···C(23)	3.231 (2)
C(22)···C(20)	2.411 (2)	H(18)···Cl(2)^vii^	3.565 (1)
C(22)···H(10)^ii^	3.042 (2)	H(18)···C(24)	3.210 (2)
C(22)···H(17)	2.027 (2)	H(18)···C(20)	3.272 (2)
C(22)···H(19)	2.010 (2)	H(18)···C(21)	2.040 (2)
C(22)···H(20)	3.210 (2)	H(18)···C(23)	1.999 (2)
C(20)···S(3)	2.674 (1)	H(19)···Cl(1)^vii^	3.210 (2)
C(20)···C(24)	2.398 (1)	H(19)···C(25)	3.233 (2)
C(20)···C(22)	2.411 (2)	H(19)···C(24)	2.020 (3)
C(20)···C(23)	2.779 (2)	H(19)···C(22)	2.010 (2)
C(20)···H(10)^ii^	3.057 (2)	H(19)···C(21)	3.239 (2)
C(20)···H(15)	2.019 (2)	H(20)···C(25)	2.030 (2)
C(20)···H(16)	2.028 (2)	H(20)···C(22)	3.210 (2)
C(20)···H(17)	2.040 (2)	H(20)···C(20)	3.256 (1)
C(20)···H(18)	3.272 (2)	H(20)···C(23)	2.007 (2)
C(20)···H(20)	3.256 (1)	H(21)···C(19)	2.643 (1)
C(20)···H(21)	2.023 (1)	H(21)···C(24)	2.009 (2)
C(21)···S(3)	3.284 (1)	H(21)···C(20)	2.023 (1)
C(21)···C(19)	2.526 (2)	H(21)···C(21)	3.231 (2)
C(21)···C(25)	2.366 (2)	H(21)···C(23)	3.223 (2)
C(21)···C(24)	2.738 (1)	H(21)···C(8)	3.566 (2)
C(21)···C(23)	2.380 (2)	H(21)···C(9)	3.181 (2)
C(21)···H(9)^ii^	3.232 (1)		
			
C(4)—S(1)—C(5)	103.34 (5)	C(8)—C(7)—H(3)	119.5 (2)
C(12)—S(2)—C(4)	102.13 (5)	C(8)—C(7)—C(6)	121.1 (1)
C(2)—S(3)—C(19)	102.76 (5)	H(3)—C(7)—C(6)	119.4 (1)
N(1)—C(3)—C(2)	111.76 (9)	C(18)—C(13)—C(14)	119.4 (1)
N(1)—C(3)—C(4)	120.85 (7)	C(18)—C(13)—C(12)	119.99 (8)
C(2)—C(3)—C(4)	127.4 (1)	C(14)—C(13)—C(12)	120.55 (9)
O(1)—N(1)—O(2)	124.0 (1)	C(10)—C(11)—H(7)	119.5 (1)
O(1)—N(1)—C(3)	118.61 (9)	C(10)—C(11)—C(6)	120.8 (1)
O(2)—N(1)—C(3)	117.2 (1)	H(7)—C(11)—C(6)	119.7 (1)
C(5)—C(6)—C(7)	120.45 (9)	C(21)—C(22)—C(23)	120.4 (2)
C(5)—C(6)—C(11)	121.1 (1)	C(21)—C(22)—H(18)	120.7 (1)
C(7)—C(6)—C(11)	118.5 (1)	C(23)—C(22)—H(18)	118.9 (2)
C(1)—C(2)—S(3)	120.32 (7)	C(21)—C(20)—C(19)	122.12 (9)
C(1)—C(2)—C(3)	122.36 (9)	C(21)—C(20)—C(25)	117.5 (1)
S(3)—C(2)—C(3)	116.89 (7)	C(19)—C(20)—C(25)	120.4 (1)
C(13)—C(12)—H(8)	107.6 (1)	H(17)—C(21)—C(22)	119.4 (2)
C(13)—C(12)—H(9)	109.7 (1)	H(17)—C(21)—C(20)	120.0 (2)
C(13)—C(12)—S(2)	111.76 (9)	C(22)—C(21)—C(20)	120.6 (1)
H(8)—C(12)—H(9)	109.5 (2)	H(19)—C(23)—C(24)	120.2 (2)
H(8)—C(12)—S(2)	108.71 (9)	H(19)—C(23)—C(22)	120.0 (2)
H(9)—C(12)—S(2)	109.6 (1)	C(24)—C(23)—C(22)	119.8 (1)
S(1)—C(4)—S(2)	120.88 (7)	C(17)—C(18)—H(14)	120.0 (1)
S(1)—C(4)—C(3)	116.92 (7)	C(17)—C(18)—C(13)	120.9 (1)
S(2)—C(4)—C(3)	122.11 (8)	H(14)—C(18)—C(13)	119.2 (1)
H(1)—C(5)—H(2)	109.5 (2)	C(9)—C(8)—H(4)	119.9 (2)
H(1)—C(5)—S(1)	109.6 (1)	C(9)—C(8)—C(7)	119.9 (2)
H(1)—C(5)—C(6)	109.8 (1)	H(4)—C(8)—C(7)	120.3 (2)
H(2)—C(5)—S(1)	107.4 (1)	C(15)—C(14)—H(10)	120.9 (2)
H(2)—C(5)—C(6)	107.1 (1)	C(15)—C(14)—C(13)	118.3 (1)
S(1)—C(5)—C(6)	113.40 (8)	H(10)—C(14)—C(13)	120.8 (1)
C(20)—C(19)—H(15)	109.4 (2)	C(9)—C(10)—H(6)	119.9 (2)
C(20)—C(19)—H(16)	110.1 (1)	C(9)—C(10)—C(11)	119.7 (1)
C(20)—C(19)—S(3)	106.89 (9)	H(6)—C(10)—C(11)	120.4 (2)
H(15)—C(19)—H(16)	109.5 (2)	H(5)—C(9)—C(8)	120.7 (2)
H(15)—C(19)—S(3)	110.4 (1)	H(5)—C(9)—C(10)	119.1 (2)
H(16)—C(19)—S(3)	110.6 (1)	C(8)—C(9)—C(10)	120.1 (1)
C(24)—C(25)—C(20)	121.6 (1)	C(16)—C(15)—H(11)	118.6 (2)
C(24)—C(25)—H(21)	118.7 (1)	C(16)—C(15)—C(14)	120.7 (2)
C(20)—C(25)—H(21)	119.7 (1)	H(11)—C(15)—C(14)	120.7 (2)
Cl(2)—C(1)—Cl(1)	113.58 (6)	C(17)—C(16)—H(12)	118.8 (3)
Cl(2)—C(1)—C(2)	123.70 (7)	C(17)—C(16)—C(15)	121.2 (2)
Cl(1)—C(1)—C(2)	122.70 (8)	H(12)—C(16)—C(15)	120.0 (2)
C(23)—C(24)—H(20)	119.0 (2)	H(13)—C(17)—C(18)	120.1 (2)
C(23)—C(24)—C(25)	120.2 (1)	H(13)—C(17)—C(16)	120.3 (2)
H(20)—C(24)—C(25)	120.9 (2)	C(18)—C(17)—C(16)	119.6 (2)
			
C(5)—S(1)—C(4)—S(2)	−43.15 (6)	C(24)—C(25)—C(20)—C(19)	179.43 (9)
C(5)—S(1)—C(4)—C(3)	140.26 (6)	C(24)—C(25)—C(20)—C(21)	−0.4 (1)
C(4)—S(1)—C(5)—C(6)	−81.53 (9)	H(21)—C(25)—C(20)—C(19)	−0.0 (2)
C(4)—S(1)—C(5)—H(1)	41.6 (1)	H(21)—C(25)—C(20)—C(21)	−179.9 (1)
C(4)—S(1)—C(5)—H(2)	160.37 (9)	C(25)—C(24)—C(23)—C(22)	1.5 (2)
C(4)—S(2)—C(12)—C(13)	−92.08 (6)	C(25)—C(24)—C(23)—H(19)	−178.9 (1)
C(4)—S(2)—C(12)—H(8)	26.5 (1)	H(20)—C(24)—C(23)—C(22)	−177.4 (1)
C(4)—S(2)—C(12)—H(9)	146.1 (1)	H(20)—C(24)—C(23)—H(19)	2.2 (2)
C(12)—S(2)—C(4)—S(1)	−49.13 (5)	C(6)—C(7)—C(8)—C(9)	−0.4 (3)
C(12)—S(2)—C(4)—C(3)	127.28 (7)	C(6)—C(7)—C(8)—H(4)	−179.5 (2)
C(19)—S(3)—C(2)—C(3)	36.39 (9)	H(3)—C(7)—C(8)—C(9)	179.8 (2)
C(19)—S(3)—C(2)—C(1)	−150.95 (9)	H(3)—C(7)—C(8)—H(4)	0.7 (3)
C(2)—S(3)—C(19)—C(20)	−168.16 (7)	C(12)—C(13)—C(18)—C(17)	−176.9 (2)
C(2)—S(3)—C(19)—H(15)	−49.3 (2)	C(12)—C(13)—C(18)—H(14)	3.9 (3)
C(2)—S(3)—C(19)—H(16)	71.9 (1)	C(14)—C(13)—C(18)—C(17)	1.1 (2)
C(2)—C(3)—N(1)—O(2)	54.1 (1)	C(14)—C(13)—C(18)—H(14)	−178.1 (2)
C(2)—C(3)—N(1)—O(1)	−122.3 (1)	C(12)—C(13)—C(14)—C(15)	177.4 (2)
C(4)—C(3)—N(1)—O(2)	−126.7 (1)	C(12)—C(13)—C(14)—H(10)	−3.4 (3)
C(4)—C(3)—N(1)—O(1)	56.8 (1)	C(18)—C(13)—C(14)—C(15)	−0.5 (2)
N(1)—C(3)—C(2)—S(3)	67.42 (9)	C(18)—C(13)—C(14)—H(10)	178.7 (2)
N(1)—C(3)—C(2)—C(1)	−105.1 (1)	C(6)—C(11)—C(10)—C(9)	1.0 (2)
C(4)—C(3)—C(2)—S(3)	−111.7 (1)	C(6)—C(11)—C(10)—H(6)	−179.3 (2)
C(4)—C(3)—C(2)—C(1)	75.8 (1)	H(7)—C(11)—C(10)—C(9)	−178.8 (2)
N(1)—C(3)—C(4)—S(1)	171.14 (6)	H(7)—C(11)—C(10)—H(6)	1.0 (3)
N(1)—C(3)—C(4)—S(2)	−5.4 (1)	C(23)—C(22)—C(21)—C(20)	0.7 (2)
C(2)—C(3)—C(4)—S(1)	−9.9 (1)	C(23)—C(22)—C(21)—H(17)	179.3 (1)
C(2)—C(3)—C(4)—S(2)	173.60 (7)	H(18)—C(22)—C(21)—C(20)	−179.4 (2)
C(7)—C(6)—C(5)—S(1)	98.1 (1)	H(18)—C(22)—C(21)—H(17)	−0.8 (2)
C(7)—C(6)—C(5)—H(1)	−24.8 (2)	C(21)—C(22)—C(23)—C(24)	−1.6 (2)
C(7)—C(6)—C(5)—H(2)	−143.6 (1)	C(21)—C(22)—C(23)—H(19)	178.8 (2)
C(11)—C(6)—C(5)—S(1)	−82.6 (1)	H(18)—C(22)—C(23)—C(24)	178.5 (2)
C(11)—C(6)—C(5)—H(1)	154.5 (2)	H(18)—C(22)—C(23)—H(19)	−1.1 (2)
C(11)—C(6)—C(5)—H(2)	35.7 (2)	C(19)—C(20)—C(21)—C(22)	−179.5 (1)
C(5)—C(6)—C(7)—C(8)	−179.2 (1)	C(19)—C(20)—C(21)—H(17)	1.9 (2)
C(5)—C(6)—C(7)—H(3)	0.6 (2)	C(25)—C(20)—C(21)—C(22)	0.3 (2)
C(11)—C(6)—C(7)—C(8)	1.4 (2)	C(25)—C(20)—C(21)—H(17)	−178.3 (1)
C(11)—C(6)—C(7)—H(3)	−178.7 (2)	C(13)—C(18)—C(17)—C(16)	−1.1 (3)
C(5)—C(6)—C(11)—C(10)	179.0 (1)	C(13)—C(18)—C(17)—H(13)	179.0 (3)
C(5)—C(6)—C(11)—H(7)	−1.2 (2)	H(14)—C(18)—C(17)—C(16)	178.1 (2)
C(7)—C(6)—C(11)—C(10)	−1.7 (2)	H(14)—C(18)—C(17)—H(13)	−1.8 (4)
C(7)—C(6)—C(11)—H(7)	178.1 (2)	C(7)—C(8)—C(9)—C(10)	−0.3 (3)
S(3)—C(2)—C(1)—Cl(2)	−172.16 (8)	C(7)—C(8)—C(9)—H(5)	178.3 (2)
S(3)—C(2)—C(1)—Cl(1)	6.0 (2)	H(4)—C(8)—C(9)—C(10)	178.8 (2)
C(3)—C(2)—C(1)—Cl(2)	0.1 (2)	H(4)—C(8)—C(9)—H(5)	−2.6 (4)
C(3)—C(2)—C(1)—Cl(1)	178.23 (9)	C(13)—C(14)—C(15)—C(16)	0.1 (3)
S(2)—C(12)—C(13)—C(18)	65.4 (1)	C(13)—C(14)—C(15)—H(11)	179.3 (3)
S(2)—C(12)—C(13)—C(14)	−112.5 (1)	H(10)—C(14)—C(15)—C(16)	−179.1 (3)
H(8)—C(12)—C(13)—C(18)	−53.9 (2)	H(10)—C(14)—C(15)—H(11)	0.1 (4)
H(8)—C(12)—C(13)—C(14)	128.2 (2)	C(11)—C(10)—C(9)—C(8)	0.1 (3)
H(9)—C(12)—C(13)—C(18)	−172.9 (2)	C(11)—C(10)—C(9)—H(5)	−178.6 (2)
H(9)—C(12)—C(13)—C(14)	9.2 (2)	H(6)—C(10)—C(9)—C(8)	−179.7 (2)
S(3)—C(19)—C(20)—C(25)	−108.2 (1)	H(6)—C(10)—C(9)—H(5)	1.6 (3)
S(3)—C(19)—C(20)—C(21)	71.6 (1)	C(14)—C(15)—C(16)—C(17)	−0.1 (4)
H(15)—C(19)—C(20)—C(25)	132.3 (1)	C(14)—C(15)—C(16)—H(12)	−178.4 (3)
H(15)—C(19)—C(20)—C(21)	−47.8 (2)	H(11)—C(15)—C(16)—C(17)	−179.4 (3)
H(16)—C(19)—C(20)—C(25)	12.0 (2)	H(11)—C(15)—C(16)—H(12)	2.3 (5)
H(16)—C(19)—C(20)—C(21)	−168.2 (1)	C(15)—C(16)—C(17)—C(18)	0.7 (4)
C(20)—C(25)—C(24)—C(23)	−0.5 (2)	C(15)—C(16)—C(17)—H(13)	−179.5 (3)
C(20)—C(25)—C(24)—H(20)	178.4 (1)	H(12)—C(16)—C(17)—C(18)	179.0 (3)
H(21)—C(25)—C(24)—C(23)	179.0 (1)	H(12)—C(16)—C(17)—H(13)	−1.2 (5)
H(21)—C(25)—C(24)—H(20)	−2.2 (2)		

Symmetry codes: (i) −*x*+1, −*y*−1, −*z*; (ii) −*x*+1, −*y*, −*z*; (iii) *x*, *y*−1, *z*; (iv) −*x*, −*y*, −*z*; (v) *x*+1, *y*, *z*; (vi) *x*+1, *y*−1, *z*; (vii) *x*, *y*+1, *z*.
